# Exposure rate of needlestick and sharps injuries among Australian veterinarians

**DOI:** 10.1186/1745-6673-4-25

**Published:** 2009-08-28

**Authors:** Peter A Leggat, Derek R Smith, Richard Speare

**Affiliations:** 1 Anton Breinl Centre for Public Health and Tropical Medicine, James Cook University, Townsville, Queensland, 4811, Australia; 2 WorkCover New South Wales Research Centre of Excellence, Faculty of Health, University of Newcastle, Ourimbah, New South Wales, 2258, Australia

## Abstract

**Background:**

Needlestick and sharps injuries (NSI) represent an important occupational health issue in veterinary practice. Little is known about the distribution and correlates of NSI among Australian veterinarians.

**Methods:**

A questionnaire-based NSI survey was mailed to 1094 veterinarians registered with the Veterinary Surgeons Board of Queensland during 2006.

**Results:**

A total of 664 surveys were returned from 1038 eligible participants (response rate 64.0%) with 56.8% being male, around one-third in the >50 years age group and about half aged 31-50 years. Just over two-fifths were working in small animal practice only. Around three quarters (75.3%) reported suffering at least one NSI in the previous 12 months, while 58.9% reported suffering from at least one contaminated NSI during the previous 12 months, which crudely extrapolates to an exposure rate of 75.3 and 58.9 NSI per 100 person-years respectively. Risk factors for contaminated NSI were female gender, working in small or mixed animal practice, being less experienced, seeing more patients per week and working longer hours per week. The most common causative devices were syringes (63.7%), suture needles (50.6%) and scalpel blades (34.8%).

**Conclusion:**

The exposure rate of NSI is high for Queensland veterinarians and clearly remains a major occupational health problem. Current guidelines and strategies to reduce NSI in veterinary practice should be promoted, but appear to be adapted from human health care. Studies to understand why veterinarians have such high NSI rates are required to not only identify risk factors for NSI, but also to determine attitudes and beliefs about NSI. From these studies specific strategies for veterinarians can be designed and trialed to develop evidence-based guidelines and policies that are effective in decreasing the exposure rate of NSI in veterinary practice.

## Introduction

Needlestick and sharps injuries (NSI) represent an important occupational health and safety issue in contemporary health care practice. Communicable diseases transmitted by NSI that commonly trouble health care workers (HCW), such as viral hepatitis and Human Immunodeficiency Virus, are of little concern for veterinarians, except those dealing with non-human primates [1]. Although zoonotic diseases are common, there are few pathogens of domestic animals that are transmitted to humans by the blood borne route. *Bartonella *spp. in cats appears to be the only zoonotic pathogen that causes chronic and recurrent bacteraemias in animals [2]. Transmission of *B. henselae *to a human via the bite of an infected cat flea highlighted that transmission by NSI could occur in theory [3], but no cases have been reported after NSI in small animal practice. Reports of communicable diseases acquired by NSI in veterinarians or animal attendants are rare. NSI was not a risk factor for seropositivity for leptospirosis in veterinarians in USA [4]. We could locate only two reports in veterinary practice where a NSI resulted in a zoonotic disease: Herpesvirus simiae from a Rhesus monkey that infected an animal attendant after a NSI [5] and blastomycosis that developed in a veterinarian after a NSI associated with a fine needle aspiration [6]. Although the recent NSI of a veterinarian while euthanizing a horse positive for Hendra virus, a PC4 emerging infectious disease agent with a high case fatality rate, highlighted the potential zoonotic risks that veterinarians now face [7], blood borne transmission of zoonotic pathogens have been considered a minor hazard for veterinarians.

Hence, in veterinary practice the emphasis on hazard from NSI has been on accidental injection of manufactured biologicals and parenteral drugs (including antibiotics, chemotherapeutics, euthanasia solutions, tranquilisers, and anaesthetic agents) rather than infectious agents from animals [1]. The major reason for this is that veterinary patients are far less compliant than human patients and movement of the animal at the time of needle puncture is common. This increases the risk of NSI for the veterinarian and the chances that syringes will contain therapeutic substances. In a survey of Wisconsin veterinarians injecting cattle, 63% of NSI occurred during injection [8], while a study on HCW found only 39% of NSI occurred during the procedure [9]. Examples of biologicals that veterinarians are particularly concerned about include Strain 19 and RB51 vaccines (live attenuated *Brucella abortus *for the control of bovine brucellosis) and Johne's vaccine (killed *Mycobacterium paratuberculosis*) for the control of Johne's disease in cattle and sheep as well as other vaccines with a similar oily adjuvant. A Canadian survey of veterinarians who vaccinated calves with Strain 19 found 46% had injected themselves at least once and of these at least 45% developed moderate to severe reactions [10]. There is one report of a veterinarian who apparently died after a NSI with Strain 19 [11]. For RB51, accidental inoculations have been reported with local or systemic reactions [12,13]. For Johne's disease vaccine 9.5% of Wisconsin veterinarians using the vaccine had accidentally inoculated themselves and 26% of NSI caused reactions [8]. Surgical debridement to remove the oily adjuvant is now recommended after injection of Johne's disease vaccine and other vaccines with a similar adjuvant [14]. Vaccines were involved in 40% of NSI in zoo veterinarians in the USA [15].

The other major concern veterinarians have about NSI is accidental injection of pharmaceutical compounds, in particular, drugs used to immobilise large animals [16]. Etorphine™, a very potent injectable synthetic narcotic used in large animals as Immobilon™, was so potent that even accidental application to skin was potentially fatal [17]. Immobilon™ was perceived as such a risk that after several death and near-death episodes involving veterinarians, its use was severely restricted by legislation [18]. Hormones from NSI can also be a hazard; a female veterinarian spontaneously aborted after a NSI with a prostaglandin [19]. Other drugs have been involved in reactions after NSI including fentanyl [16] and tilmicosin [20]. Within the veterinary profession since the emphasis has been less on NSI *per se*, and more on the potential effects of the syringe contents, the prevention of NSI was for many years a much lower priority than in HCW.

NSI has not been widely investigated in the veterinary profession and only a few studies report incidence (NSI per time per person) or exposure rate with most reporting life-time prevalence. In one survey of female veterinarians in USA, 64% reported one or more NSI during their careers, with a reported incidence of 9.3 NSI per 100 person-years [19]. Vaccines were associated with 50% of the incidents in that study [19]. The incidence of NSI in the survey of Wisconsin veterinarians vaccinating cattle with Johne's disease vaccine was 5.5 NSI per 100 person-years [8]. A survey of 735 veterinarians in USA found a much higher rate of 0.45 NSI per respondent over three years, a rate of 15 NSI per 100 person-years [20]. Incidence of NSI in veterinarians attending a national conference in USA was 74.2 per 100 person-years [4]. In another study examining zoo veterinarians in the United States, 87% reported one or more NSI with 6.5% requiring medical treatment [15]. In this study, more than half of respondents reported NSI associated with animal blood, antimicrobials and vaccines [15]. In a Western Australian study of veterinary practices, 71% of respondents had been injured over a 10 year period with the most common injuries being dog and cat bites, cat scratches and NSI [21]. The study did not specify the prevalence of NSI. In an Australian study of veterinary nurses 71% reported NSI with about two-thirds of these associated with injection of various substances, including antibiotics (13%), euthanasia chemicals (11%), sedatives (9%), vaccines (8%) and anaesthetics (8%) [22].

Although there is now a greater emphasis in veterinary practice in Australia on prevention of NSI, little is known about the current exposure rate of NSI among Australian veterinarians [1]. Therefore, the current study was undertaken to investigate NSI among veterinarians registered in Queensland, Australia.

## Methods

This study involved a cross-sectional epidemiological analysis of NSI among all veterinarians who were registered with the Veterinary Surgeons Board of Queensland during 2006. Data was obtained by means of a mailed self-reporting questionnaire. A three-page, A4-sized, self-reporting questionnaire was subsequently mailed to each veterinarian, including an information sheet and a reply paid envelope. Two reminders were sent. All information was collected anonymously, and informed consent was implied when veterinarians completed and returned their questionnaires. Our three-page survey included questions such as age, sex, years in practice, type of practice, number of patients seen per day, occurrence of NSI over the past 12 months, whether their injuries involved contaminated devices (previously used on a patient), as well as the types of devices causing the NSI. Data was analysed by statistical software [23] to establish the exposure rate of NSI as well as demographic correlates and possible risk factors. Differences in NSI exposure rates by sex and age (P for Trend) were evaluated using the Chi-square test. *P *values below 0.05 were regarded as statistically significant throughout all analyses. Ethical clearance for this study was obtained from the James Cook University Human Research Ethics Committee (No. H2465).

## Results

### Demographics

A total of 664 surveys were returned from 1038 eligible veterinarians, giving a response rate of 64.0%. Demographic data is presented in Table 1.

**Table 1:** Demographic Characteristics

	**Female**	**Male**	**Total**	
**Demographics**	**(%)**	**(%)**	**(%)**	**P value**^a^
				
**Age **(years)				
<30	(24.9)	(6.1)	(14.2)	
31-40	(36.7)	(16.6)	(25.7)	
41-50	(28.5)	(23.4)	(25.6)	<0.001
>50	(10)	(52.9)	(34.6)	
				
**Type of Practice**^b^				
Small animal	(52.5)	(36.6)	(43.4)	<0.001
Large animal	(2.8)	(10.6)	(7.3)	<0.001
Mixed animal	(32.7)	(30.8)	(31.6)	0.324
Specialist	(7.0)	(8.2)	(7.7)	0.341
Non-clinical	(4.9)	(7.7)	(7.7)	0.013
University	(6.3)	(4.3)	(5.2)	0.152
Other	(1.4)	(6.4)	(4.2)	<0.001

^a^P for Trend evaluated using the chi square test by sex, except for age;

^b^Percentages for type of practice are percentages within sex.

Summary background data on age, number of years in clinical practice, working hours per week, and number of patients treated per day have been summarised by sex in Table 2. Of the respondents 56.8% were male, about one-third were >50 years and about half were aged 31-50 years. Just over two-fifths were working in small animal practice only. Female veterinarians were significantly younger (p < 0.001) than their male counterparts.

**Table 2:** Demographic and workplace items versus NSI exposure rate

	**Any NSI**		**Contaminated NSI**	
	**Numbers (per 100 person-years)**	P value^a^	**Numbers (per 100 person-years)**	P value^a^

**Hours per week**				
<20	(55.6)	P < 0.01	(44.4)	P < 0.05
20-30	(74.5)		(50.9)	
31-50	(80.1)		(62.2)	
>50	(75.8)		(61.6)	
				
**Patients per week**				
<20	(50.0)	P < 0.001	(35.6)	P < 0.001
20-30	(82.0)		(59.6)	
31-50	(83.2)		(68.8)	
>50	(86.6)		(68.3)	
				
**Years as veterinarian**				
<5	(89.9)	P < 0.001*	(70.8)	V
5-10	(80.2)		(65.9)	
11-20	(79.9)		(64.9)	
>20	(66.9)		(50.0)	
				
**Type of Practice**^b^				
Small animal	(83.6)	P < 0.001	(63.4)	P < 0.05
Large animal	(58.3)	P < 0.01*	(37.5)	P < 0.01*
Mixed animal	(86.6)	P < 0.001	(75.6)	P < 0.001
Specialist	(70.6)	P = 0.420	(51.0)	P = 0.233
Non-clinical	(37.5)	P < 0.001*	(25.0)	P < 0.001*
University	(41.2)	P < 0.001*	(26.5)	P < 0.001*
Other	(31.0)	P < 0.001*	(17.2)	P < 0.001*

^a ^Chi square for trend;

^b^Percentages for type of practice are percentages of veterinarians reporting an NSI or contaminated NSI within these categories.

* Significantly lower reported prevalence of NSI or contaminated NSI during the previous 12 months

### NSI Prevalence

Around three-quarters (75.3 per cent) of veterinarians reported at least one NSI in the previous 12-months and 58.9% reported at least one contaminated NSI during the previous 12 months. Females were significantly more likely to report having an NSI (82.6% verses 69.8%; p < 0.001) and a contaminated NSI (66.0% verses 53.7%; p < 0.01) in the previous 12 months. Table 2 indicates NSI and contaminated NSI exposure rates by hours of work per week, patients per week, years in practice and by type of practice.

### Causative Device

The prevalence of various devices causing NSI is stratified by sex in Table 3. The most common causative devices were syringes (63.7%), suture needles (50.6%) and scalpel blades (34.8%).

**Table 3:** Prevalence of NSI causative device in the previous 12 months.

	**Female** **(%)**	**Male** **(%)**	**Total** **(%)**	**P value**^a^
**Syringe**				
Clean	(23.8)	(18.2)	(20.6)	P < 0.001
Used	(22.7)	(17.6)	(19.8)	
Both	(25.2)	(21.9)	(23.3)	
				
**Scalpel**				
Clean	(11.3)	(7.8)	(9.3)	P = 0.196
Used	(14.9)	(16.3)	(15.7)	
Both	(11.7)	(8.3)	(9.8)	
				
**Suture needles**				
Clean	(6.0)	(7.8)	(7.0)	P < 0.01
Used	(36.9)	(28.1)	(31.9)	
Both	(14.2)	(9.9)	(11.7)	
				
**Ampoule/Vial**				
Clean	(11.7)	(5.6)	(8.2)	P < 0.01
Used	(2.1)	(1.1)	(1.5)	
Both	(1.1)	(0.5)	(0.8)	
				
**Other Sharps**				
Clean	(2.5)	(0.0)	(1.1)	P < 0.01
Used	(2.8)	(5.3)	(4.3)	
Both	(1.8)	(1.6)	(1.7)	

^a^Chi Square examining combined "used" and "both" categories

## Discussion

This cross-sectional study examined the exposure rate and other epidemiological characteristics of self-reported NSI. The response rate for our study was relatively high (64%), probably due to the interest in the topic, the short questionnaire itself and the follow-ups conducted. One of the major limitations of this type of study is that what people report may differ from their actual situation. Since we selected all veterinarians registered in Queensland, our final cohort of veterinarians is a reasonably comprehensive sample for that state. We assume that since the veterinary profession in Australia is reasonably homogenous, this survey will also reflect the situation in Australia as a whole, but we have no evidence for this assumption.

The exposure rate of NSI reported by Queensland veterinarians is high, if we extrapolate the crude 12 month exposure (75.3 per 100 person-years). However, it is similar to the exposure rate of 74.2 NSI per 100 person-years from a recent USA study [4]. These exposure rates are higher than previous studies on veterinarians that ranged between 5.5-15 NSI per 100 person-years [8,19,24]. The exposure rate of NSI in Queensland veterinarians was, however, much higher than that reported amongst dentists and nurses, using a similar methodology in the same region, if we extrapolate the same crude 12 month exposure (27.7 and 17.7 NSI per 100 person-years respectively) [25,26]. It is also much higher than the prevalence rates described for physicians in USA (4-23%) [27] and HCW in UK (0.8-5 NSI per 100 person-years) [28]. Little has been published concerning the risk factors for NSI in veterinarians [1]. The findings that large animal practice was associated with a significantly lower exposure rate of NSI and that small animal and mixed practice was associated with a significantly higher exposure rate of NSI are consistent with that observed in a study of female veterinarians graduated from US colleges [19]. The finding of a significant trend towards a higher incidence of NSI amongst less experienced veterinarians is consistent with that observed in a study of personnel in two non-human primate laboratories in the USA [29].

The high exposure rate of NSI revealed during this study suggest that is very important that veterinarians observe basic measures for reducing NSI, which are discussed elsewhere [1], and examine the possibility of using safer devices [1]. About two thirds of the "sharps" injuries were contaminated "sharps" or NSI; however, one third were non-contaminated. As such, it is important that veterinarians remain vigilant for these types of exposures, so that devices contaminated by the veterinarians themselves do not subsequently create a downstream hazard for other veterinary practice staff.

Our study showed that the most common "sharps" injuries and contaminated "sharps" injuries among veterinarians were from syringes, suture needles and scalpels, which is consistent with previous studies from Western Australia and other countries [15,19,21]. Of concern with these NSI is the fact that they often occur while giving injections or operating on the patient, when there is most likely to be some residual pharmaceutical agent or bodily fluid in the needle or on the instrument. It is important that the Australian Veterinary Association's Code for Infection Control [30] is adhered to in relation to preventing NSI and following any "sharps" injury during veterinary practice [30]; however, the AVA Code lacks details. Although prevention of NSI has been previously identified as an area needing more effective management among veterinary personnel [1], it appears that current guidelines to reduce NSI in veterinary practice are not based on veterinary evidence, but are adapted from studies in human health care.

## Conclusion

The exposure rate of NSI is high for Queensland veterinarians and clearly remains a major occupational health problem. Current guidelines and strategies to reduce NSI in veterinary practice should be promoted, but appear to be adapted from human health care. Studies to understand why veterinarians have such high NSI rates are required to not only identify risk factors for NSI, but also to determine attitudes and beliefs about NSI. From these studies specific strategies for veterinarians can be designed and trialed to develop evidence-based guidelines and policies that are effective in decreasing the exposure rate of NSI in veterinary practice.

## Competing interests

The authors declare that they have no competing interests.

## Authors' contributions

PAL conceived of the study and gathered the data. PAL, DRS and RS drafted the manuscript. PAL performed the statistical analysis. All authors read and approved the manuscript.
